# Real-Time Visualization and Quantification of Human Cytomegalovirus Replication in Living Cells Using the ANCHOR DNA Labeling Technology

**DOI:** 10.1128/JVI.00571-18

**Published:** 2018-08-29

**Authors:** Bernard Mariamé, Sandrine Kappler-Gratias, Martin Kappler, Stéphanie Balor, Franck Gallardo, Kerstin Bystricky

**Affiliations:** a Laboratoire de Biologie Moléculaire Eucaryote (LBME), Centre de Biologie Intégrative (CBI), University of Toulouse, CNRS, UPS, Toulouse, France; b Institute for Advanced Life Science Technology (ITAV), University of Toulouse, CNRS, UPS, Toulouse, France; c statalpha, Baziège, France; d Multiscale Electron Imaging (METi) Facility, Centre de Biologie Integrative (CBI), University of Toulouse, CNRS, UPS, Toulouse, France; e NeoVirTech SAS, Toulouse, France; University of Southern California

**Keywords:** HCMV, antiviral screening, fluorescent virus, live imaging, viral replication

## Abstract

Human cytomegalovirus (HCMV) induces latent lifelong infections in all human populations. Between 30% and nearly 100% of individuals are affected depending on the geographic area and socioeconomic conditions. The biology of the virus is difficult to explore due to its extreme sophistication and the lack of a pertinent animal model. Here, we present the first application of the ANCHOR DNA labeling system to a herpesvirus, enabling real-time imaging and direct monitoring of HCMV infection and replication in living human cells. The ANCHOR system is composed of a protein (OR) that specifically binds to a short, nonrepetitive DNA target sequence (ANCH) and spreads onto neighboring sequences by protein oligomerization. When the OR protein is fused to green fluorescent protein (GFP), its accumulation results in a site-specific fluorescent focus. We created a recombinant ANCHOR-HCMV harboring an ANCH target sequence and the gene encoding the cognate OR-GFP fusion protein. Infection of permissive cells with ANCHOR-HCMV enables visualization of nearly the complete viral cycle until cell fragmentation and death. Quantitative analysis of infection kinetics and of viral DNA replication revealed cell-type-specific HCMV behavior and sensitivity to inhibitors. Our results show that the ANCHOR technology provides an efficient tool for the study of complex DNA viruses and a new, highly promising system for the development of innovative biotechnology applications.

**IMPORTANCE** The ANCHOR technology is currently the most powerful tool to follow and quantify the replication of HCMV in living cells and to gain new insights into its biology. The technology is applicable to virtually any DNA virus or viruses presenting a double-stranded DNA (dsDNA) phase, paving the way to imaging infection in various cell lines, or even in animal models, and opening fascinating fundamental and applied prospects. Associated with high-content automated microscopy, the technology permitted rapid, robust, and precise determination of ganciclovir 50% and 90% inhibitory concentrations (IC_50_ and IC_90_) on HCMV replication, with minimal hands-on time investment. To search for new antiviral activities, the experiment is easy to upgrade toward efficient and cost-effective screening of large chemical libraries. Simple infection of permissive cells with ANCHOR viruses in the presence of a compound of interest even provides a first estimation of the stage of the viral cycle the molecule is acting upon.

## Abstract (precis)

The ANCHOR technology is currently the most powerful tool to follow and quantify the replication of HCMV in living cells and to gain new insights into its biology. The technology is applicable to virtually any DNA virus or viruses presenting a double-stranded DNA (dsDNA) phase, paving the way to imaging infection in various cell lines, or even in animal models, and opening fascinating fundamental and applied prospects. Associated with high-content automated microscopy, the technology permitted rapid, robust, and precise determination of ganciclovir 50% and 90% inhibitory concentrations (IC_50_ and IC_90_) on HCMV replication, with minimal hands-on time investment. To search for new antiviral activities, the experiment is easy to upgrade toward efficient and cost-effective screening of large chemical libraries. Simple infection of permissive cells with ANCHOR viruses in the presence of a compound of interest even provides a first estimation of the stage of the viral cycle the molecule is acting upon.

## INTRODUCTION

**FIG 1:**
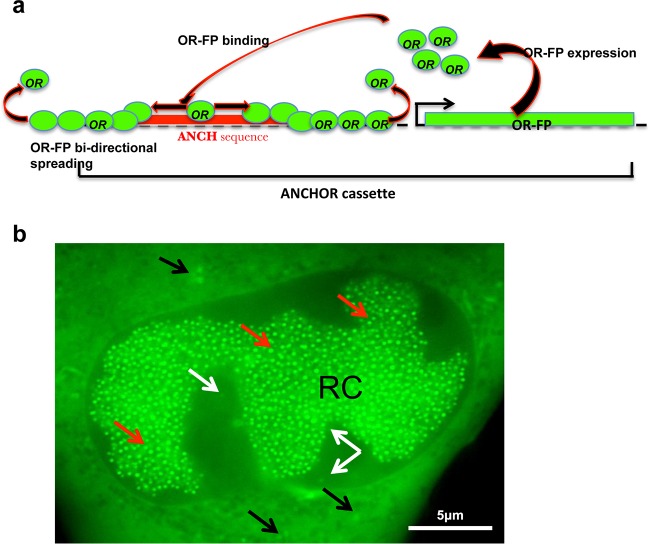
Principle of the ANCHOR DNA labeling technology. (a) The ANCHOR system is composed of an ANCH DNA target sequence less than 1 kb long (red box) that specifically binds dimers of OR protein through nucleation sites. OR dimers spread on the DNA, and more OR dimers are recruited to form a large metastable nucleoprotein complex. When OR is fused to a fluorescent marker (green circles), accumulation of this complex on the ANCH sequence forms a spot that is easily detected by fluorescence microscopy. The ANCH sequence and a chimeric gene encoding the corresponding OR protein fused to a fluorescent protein may be associated in a cassette that can be inserted in a site-specific manner into the target viral genome, as shown in Fig. 2. (b) Representative image of a TB40-ANCHOR3-infected MRC5 cell 48 h p.i. showing accumulation of fluorescent spots in the nucleus (taken with a wide-field Zeiss Axiovert Observer Z1 with a 1.4-numerical-aperture [NA] 63× objective). Scale bar, 1 μm.

Human cytomegalovirus (HCMV), also called human herpesvirus 5 (HHV5), belongs to the Betaherpesviridae family and, like all herpesviruses (HVs), is able to establish lifelong latency in infected individuals (1). HCMV is the largest HHV, with a double-stranded DNA (dsDNA) genome of about 240 kb. It is usually transmitted through body fluids, such as saliva, urine, or breast milk, but also through sexual contact (2). Primary infection is generally benign or silent in healthy individuals but may be much more serious and even life threatening in immunocompromised patients, especially those who have received hematopoietic cells or solid-organ transplants, or in AIDS patients. The virus is also able to cross the placental barrier, and primary HCMV infection during pregnancy, mainly during the first quarter, is the leading cause of birth defects, with an estimate of 1 million congenital HCMV infections worldwide per year (3, 4). Among those infected, possibly up to 25% of newborns suffer permanent sensorineural and intellectual deficits. *In vivo* infection is poorly understood but most likely initiates in mucosal tissue and then spreads through blood monocytes, which disseminate the virus. HCMV binds to heparan sulfate proteoglycan (5) and to numerous cell membrane structures, among which CD13 (6), annexin II (7), DC-SIGN (dendritic cell-specific intercellular adhesion molecule-3-grabbing nonintegrin) (8), EGFR (epidermal growth factor receptor) (9), and PDGFR-α (platelet-derived growth factor receptor alpha) (10) are candidate receptors. This may in part explain the remarkably broad cell tropism of the virus, which is able to infect and replicate in many cell types, including epithelial, dendritic, fibroblastic, endothelial, and smooth muscle cells (11), and to establish latency in CD34^+^ hematopoietic progenitor cells (12). Extensive efforts have allowed partial deciphering of the biology of this highly sophisticated virus, but much remains to be learned about *in vivo* infection kinetics. Techniques to track real-time infections in live cells have been developed for RNA viruses (13–15) and also for herpesviruses (16–18). However, until now, fluorescent tracking of HVs relied on green fluorescent protein (GFP) expression alone or on fusion of the GFP gene with a viral structural gene. These engineered viruses have greatly contributed to some pioneering work but did not provide quantitative information about replication kinetics of the viral genome. Therefore, to gain a better understanding of the fundamental biology of HVs, we have introduced a new technology enabling real-time follow-up and counting of viral genomes during infection in live cells and also possibly in live-animal models. In this paper, we present the use of the patented ANCHOR DNA labeling technology (19) for tracking of HCMV in living cells. ANCHOR is a bipartite system derived from a bacterial ParABS chromosome segregation machinery. Under its natural form in bacteria, the ParABS system consists of a short, nonrepetitive target DNA sequence containing a limited number of nucleation parS sites to which ParB proteins bind and then spread onto adjacent DNA through a mechanism of protein-protein interaction. The third component of the system is an ATPase involved in the last steps of bacterial chromosome or plasmid segregation. Under its engineered form, called ANCHOR, OR proteins (ParB) specifically bind to the cognate, shortened ANCH sequence, which comprises palindromic parS nucleation sites (20, 21). If the OR protein is fused to a fluorescent protein (FP), its accumulation on the ANCH target sequence and spread over neighboring sequences result in the formation of an easily detectable fluorescent focus, thereby identifying the position of the ANCH-tagged DNA locus (Fig. 1a). Different ANCHOR systems (1 to 4, derived from various bacteria) have been used successfully to analyze the motion of single genomic locus and DNA double-strand break processing in living Saccharomyces cerevisiae cells (22) and chromatin dynamics during transcription in human cells (23). These ANCHOR systems were shown not to perturb chromatin structure and function despite the presence of up to 500 OR proteins on and around the ANCH sequence (23). Here, we created HCMV genomes containing the ANCH2 target sequence (HCMV-ANCH2) or both the ANCH3 target sequence and the gene encoding the corresponding OR3-GFP protein (HCMV-ANCHOR3). In the latter case, OR3-GFP proteins (which do not present any known intracellular localization sequence) freely diffuse in the cell and rapidly associate with the ANCH3 sequence, rendering the HCMV DNA fluorescent and detectable by microscopy as well-defined spots over a uniform background of OR-GFP proteins. Thanks to these engineered virions, we were able to visualize early infection and initial duplication of the incoming genomes, viral DNA amplification, replication, and cell death in real time and in live cells. All these steps were simply observed by microscopic examination, with no additional manipulation and without fixation, extraction, or reagents of any kind, emphasizing the ease of use, power, and potential of the technology. Furthermore, analyzing the effect of ganciclovir on ANCHOR-HCMV infection illustrates the remarkable potential of the technology for time- and cost-effective screening of compound libraries in the search for new antiviral molecules. Its suitability for labeling any DNA virus (and possibly any virus presenting a dsDNA phase) offers unprecedented opportunities for new biotechnology applications.

(This article was submitted to an online preprint archive [24].)

## RESULTS

### Construction of ANCHOR-HCMV BACs.

**FIG 2:**
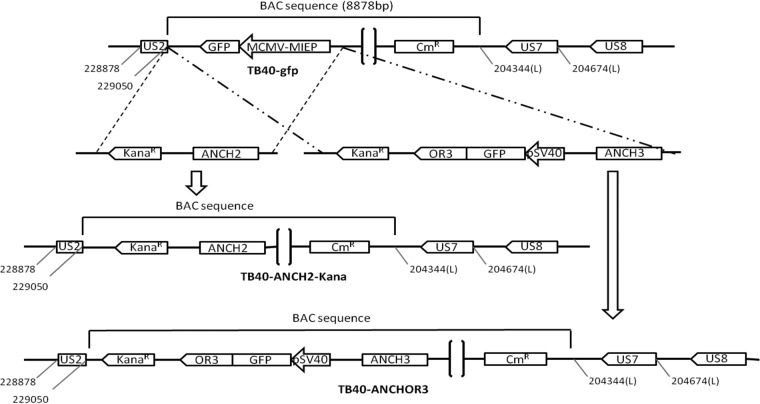
Construction of the ANCH2 or ANCHOR3 HCMV BAC used in this study and characterization of viruses derived from the TB40-ANCHOR3 HCMV BAC. Both ANCH2 and the ANCHOR3 HCMV BAC were derived from the TB40-GFP BAC, which contains the GFP gene under the control of a murine CMV immediate-early promoter (pMCMV-MIEP-GFP) inserted in the vector backbone. This gene was replaced by the desired constructs without affecting any other viral gene or sequence. The TB40-ANCH2-Kana HCMV BAC displaying a single ANCH2 target sequence was obtained by exchanging the mCMV-MIEP-GFP gene with an ANCH2-Kana^r^ cassette while the MCMV-MIEP-GFP gene was exchanged by homologous recombination with a Kana^r^-OR3/GFP-ANCH3 cassette to create the TB40-ANCHOR3 HCMV BAC.

Two ANCHOR-modified HCMV bacterial artificial chromosomes (BACs) were derived from the TB40-GFP BAC (a kind gift from E. Borst and M. Messerle). The first, TB40-ANCH2-Kana, was obtained by replacing the murine CMV immediate-early promoter (mCMV-MIEP)-GFP gene of the TB40-GFP with a single ANCH2 target sequence (together with a selection kanamycin resistance gene). The TB40-ANCHOR3 BAC was constructed in a similar way but replacing the mCMV-MIEP-GFP sequence with a cassette containing the ANCH3 target sequence, a chimeric OR3-GFP gene driven by a simian virus 40 (SV40) promoter and a kanamycin resistance gene (Fig. 2) (see Materials and Methods). Unlike the TB40-ANCH2-Kana virus, which requires separate transfection of an expression vector for its corresponding OR2 protein, TB40-ANCHOR3 is autonomous because it contains the ANCH3 target sequence and the gene encoding its cognate OR3 protein (fused to the GFP gene).

### Viruses derived from the TB40-ANCHOR-HCMV BACs are infectious.

Transfections of the purified TB40-ANCHOR BACs in MRC5 fibroblasts were poorly efficient, likely due to their very large size and the low transfection efficacy of the cells. Only sparse foci of modified cells were observed 10 days posttransfection. However, nearly 100% cytopathic effects were reached 4 to 5 weeks after transfection, indicating that the rare cells that were transfected with the TB40-ANCHOR BACs produced viruses that were fully infectious. In order to confirm infectivity and to assess whether TB40-ANCHOR3 viruses had conserved the epithelial and endothelial tropism of the original TB40 HCMV strain, TB40-ANCHOR3 viruses were used to infect new MRC5 cells, human umbilical vein endothelial cells (HUVEC), or ARPE-19 cells, which are fibroblasts, endothelial cells, and retinal epithelial cells, respectively. The three cell types became readily infected, with the appearance of fluorescence and fluorescent spots several hours postinfection (p.i.), confirming infectivity of the ANCHOR engineered viruses and the preservation of the original cellular tropism (Fig. 1b; see Fig. S1 in the supplemental material). However, infection efficacy and kinetics were clearly cell type dependent, as the numbers of infected cells obtained with identical multiplicities of infection (MOI) varied greatly from one cell type to another. For a first estimate of its replication capacity, the TB40-ANCHOR3 viral stock was titrated using two different techniques: a fluorescence assay and a classical plaque-forming assay. MRC5 cells were infected with different dilutions of the viral stock for 2 or 18 h, washed, and incubated as described above. The plate from the fluorescence assay was analyzed with an automated Arrayscan microscope 60 h p.i., while the plate from the plaque-forming assay was maintained at 37°C for 12 days and then fixed, stained, and analyzed. As shown in Table 1, very similar results were obtained with the two techniques, indicating that the viruses infecting the cells render them fluorescent and are also able to induce a complete lytic cycle. Interestingly, the contact time between cells and viruses mattered, because longer infection times systematically resulted in higher titers than shorter times, whichever technique was used. However, even if both techniques are reliable, the fluorescence technique is clearly less labor-intensive and more rapid, robust, and reproducible and hence was adopted for all subsequent titrations.

**TABLE 1:** Measurement of TB40-ANCHOR3 HCMV stock virus titers

Stock virus amt (μl)	Measured virus titer^*a*^			
	Plaque-forming assay		Fluorescence assay	
	2-h infection	18-h infection	2-h infection	18-h infection
10^−4^	6.0 × 10^7^	2.0 × 10^8^		
10^−3^	5.2 × 10^7^	1.6 × 10^8^	2.1 × 10^7^	1.2 × 10^8^
10^−2^	2.8 × 10^7^		3.9 × 10^7^	4.2 × 10^8^
10^−1^			1.2 × 10^8^	1.9 × 10^8^

a Titers were measured using a classical plaque-forming assay and a fluorescence assay. See Materials and Methods for details. The measured titers were significantly higher when infection time was increased from 2 h to 18 h. Similar results were obtained with both techniques.

### ANCHOR labeling does not interfere with viral replication rates.

**FIG 3:**
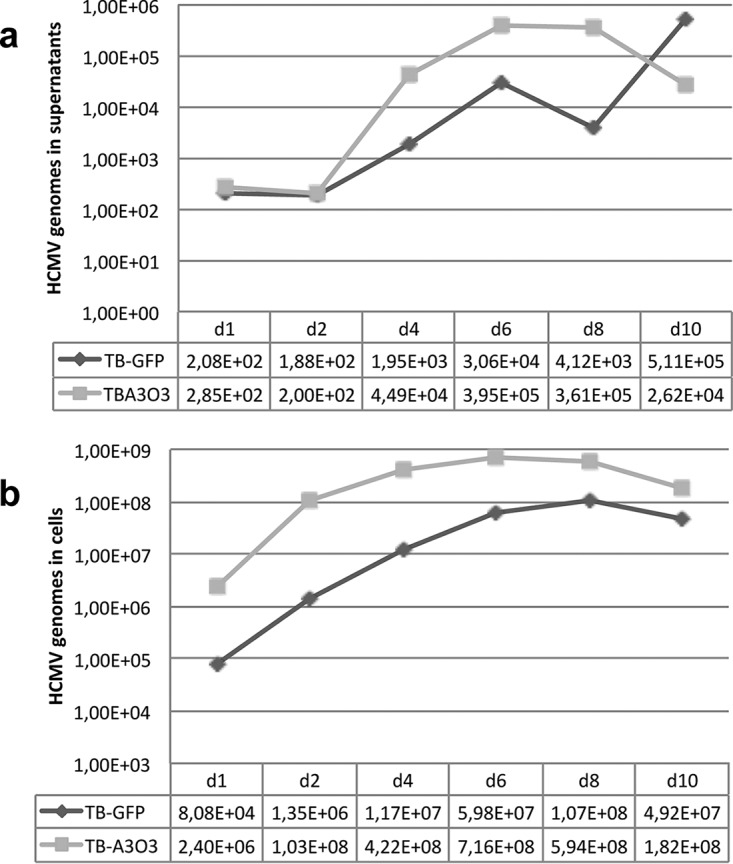
Replication curves of TB40-GFP or TB40-ANCHOR3 viruses in MRC5-infected cells. (a) Titration of HCMV genomes in the supernatants of MRC5-infected cells. (b) Titration of HCMV genomes in MRC5-infected cells. The measured values are given at the bottom of each graph and represent the total number of HCMV genomes in supernatants (a) or in cells (b).

To quantify replication kinetics more precisely, we infected MRC5 cells with TB40-GFP and TB40-ANCHOR3 viruses at an MOI of 0.2 and measured the number of viral genomes present in cells and in their supernatants until 10 days postinfection (Table 2 and Fig. 3). Both in supernatants and in cells, the number of TB40-ANCHOR3 genomes was greater than the number determined for TB40-GFP, suggesting that TB40-ANCHOR3 replicates more efficiently. In supernatants, TB40-GFP genomes were produced in quantities similar to those reported for the TB40-BAC4 parental strain (25). These results show that the presence of the ANCHOR sequences in the viral genome does not impair its replication or induce a significant functional defect.

**TABLE 2:** Replication kinetics of TB40-GFP and TB40-ANCHOR3 viruses^*a*^

Day p.i.	TB40-GFP			TB40-ANCHOR3		
	No. of cells/well (10^−3^)	No. of infected cells/well (10^−3^)	No. of viral genomes/infected cell (10^−3^)	No. of cells/well (10^−3^)	No. of cells/well (10^−3^)	No. of viral genomes/infected cell (10^−3^)
2	129/132	9.1/9	0.17/0.13	111/114	9.6/13.9	9.4/8.3
4	316/295	26.7/40.6	0.21/0.44	254/288	27.3/33.2	10.7/16.5
6	282/289	76.4/78.8	0.71/0.83	226/256	50.5/36.8	14.3/19.2
8	232/240	79.4/79.3	1.3/1.4	135/210	66.1/44.5	9.4/12.7
10	234	89.6	0.54	167	89.1	2.3

a MRC5 cells were infected at an MOI of 0.2. Cells and supernatants were harvested on days 2, 4, 6, 8, and 10 postinfection, and DNA was purified from each sample. Total numbers of viral genomes were determined for each sample using qPCR. Cells and infected cells were counted in a parallel plate. Each measurement was made in triplicate, and mean values of the two wells per time point are given for each day (except day 10 p.i.).

### Viruses derived from the TB40-ANCHOR-HCMV BAC are fluorescent and mature.

**FIG 4:**
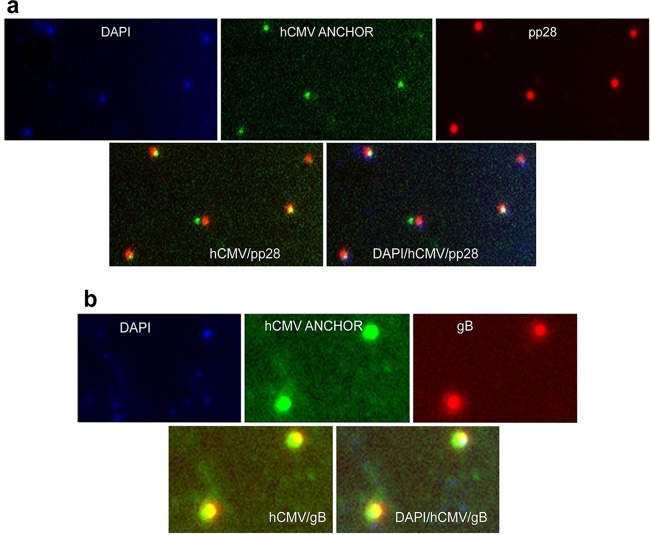
Characterization of TB40-ANCHOR3 viruses. Viral particles derived from the TB40-ANCHOR3 HCMV BAC contain DNA and OR3-GFP proteins and stain for pp28 tegument proteins (a) and for gB envelope proteins (b). They likely correspond to mature viruses with OR proteins bound to the encapsidated genomes. The images were acquired with a wide-field Zeiss Axiovert Observer Z1 with a 1.4-NA 63× objective.

TB40-ANCHOR3 viruses from the purified viral stock were immobilized on poly-l-lysine-treated glass slides and stained with Hoechst and anti-pp28 or anti-gB antibody. As shown in Fig. 4, Hoechst, GFP, anti-pp28, and anti-gB signals were perfectly superimposed, suggesting that these viral particles are mature, tegumented, and enveloped and contain DNA and OR3-GFP proteins.

### OR-GFP proteins effectively bind to ANCH sequences in ANCHOR-HCMV-infected cells.

**FIG 5:**
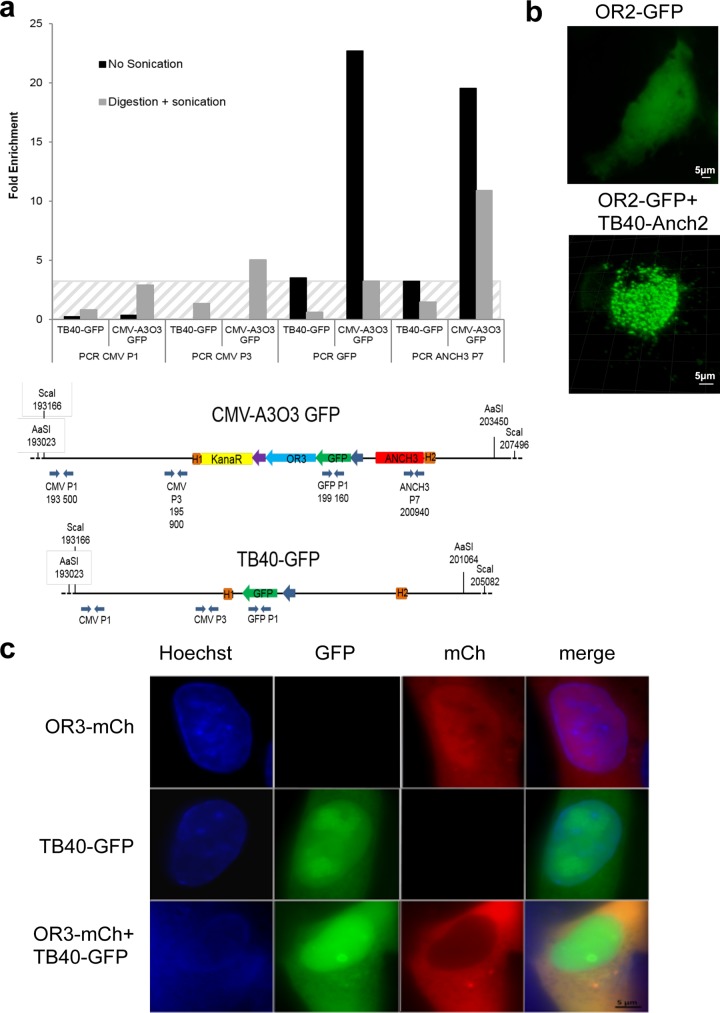
OR-FP proteins specifically bind to their target ANCH sequence, generating the spots observed in fluorescence microscopy. (a) ChIP experiment showing that chromatin extracted from TB40-ANCHOR3 HCM-infected MRC5 cells (but not from TB40-GFP-infected cells) and immunoprecipitated with anti-GFP antibody is enriched only in ANCH3 and GFP sequences; the hatched area corresponds to background noise, since no ANCH3 sequence is present in the TB40-GFP virus. (b) MRC5 cells were transfected with a single vector expressing OR2-GFP and then infected or not with TB40-ANCH2-Kana viruses; spots appear only when OR-FP and the corresponding ANCH target sequences on the viral genomes are present simultaneously in the same cell. The images were acquired 24 h posttransfection and 72 h postinfection with a wide-field Zeiss Axiovert Observer Z1 with a 1.4-NA 63× objective or a Zeiss LSM 510 NLO. (c) MRC5 cells were transfected with an expression vector for OR3-mCherry, infected with TB40-GFP, or transfected and infected simultaneously; no spots were observed in any situation, indicating that neither OR3 proteins nor TB40-GFP genomes form nonspecific spots, even when present simultaneously in the same cell. The images were acquired 24 h posttransfection and 72 h postinfection with a wide-field Zeiss Axiovert Observer Z1 with a 1.4-NA 63× objective or a Zeiss LSM 510 NLO.

Binding of OR proteins to cognate engineered ANCH target sequences was previously demonstrated in pro- and eukaryotic systems (22, 23). We confirmed that the same held true in cells infected with our HCMV-ANCHOR. For this purpose, chromatin immunoprecipitation (ChIP) experiments were performed on cells infected with TB40-GFP or TB40-ANCHOR3 viruses using antibodies against GFP. As shown in Fig. 5a, DNA immunoprecipitated from TB40-ANCHOR3-infected cells is strongly enriched in ANCH3 sequences, confirming OR3-GFP proteins bind to the ANCH3 target sequence. Enrichment in the adjacent GFP sequence suggested spreading of OR3-GFP onto neighboring DNA. No significant enrichment of more distant sequences was observed. Similarly, no significant enrichment was observed in DNA from cells infected with the TB40-GFP virus immunoprecipitated with anti-GFP antibodies.

### In infected cells, fluorescent spots result from OR-FP binding to ANCH-HCMV genomes.

To determine the nature of the observed spots (Fig. 1b; see Fig. S1 in the supplemental material), we took advantage of our TB40-ANCH2-Kana virus, which contains a single ANCH2 sequence but no OR-FP gene. When MRC5 cells were solely infected with the TB40-ANCH2-Kana viruses, no fluorescence was observed (results not shown). Transfection of an expression vector for OR2-GFP proteins in uninfected MRC5 cells resulted in uniform fluorescence in all cell compartments, while infection of OR2-GFP-transfected cells with the TB40-ANCH2-Kana virus resulted in the appearance of numerous bright spots 72 h postinfection (Fig. 5b). As a control, MRC5 cells that had been transfected with an expression vector for OR3-mCherry were infected with the TB40-GFP virus; as shown in Fig. 5c, 72 h p.i., doubly fluorescent (red and green) cells did not display any spots similar to those observed in Fig. 5b or S1, despite the fact that a nuclear structure resembling a replication compartment was clearly visible (Fig. 5c). These two experiments together demonstrate, on one hand, that OR-FP proteins or viral genomes alone do not form spots and, on the other hand, that OR-FP proteins do not form nonspecific spots on HCMV genomes. Therefore, taken together with the ChIP experiments, these results demonstrate that ANCHOR-HCMV fluorescent spots result from specific accumulation of OR-FP proteins on the corresponding ANCH sequences inserted in viral genomes.

### The ANCHOR cassette is stable in the recombinant virus.

We tested the stability of the ANCHOR phenotype after massive amplification from a single TB40-ANCHOR3 PFU up to a stock of 8 × 10^8^ infectious particles. Viruses of this stock were used to infect MRC5 cells that were fixed and stained with various anti-HCMV antibodies at different times postinfection. We found that >90% of pp28 (tegument)-positive cells were also positive for OR-GFP, revealing that, despite an amplification factor of nearly 10^9^, less than 10% of the final viruses had lost the ANCHOR phenotype (results not shown). It is noteworthy that a similar situation was also observed for the TB40-GFP stock, in which only 95% of the viruses were positive for both UL44 and GFP (results not shown).

### Real-time visualization of ANCHOR-HCMV infection in living human cells.

**FIG 6:**
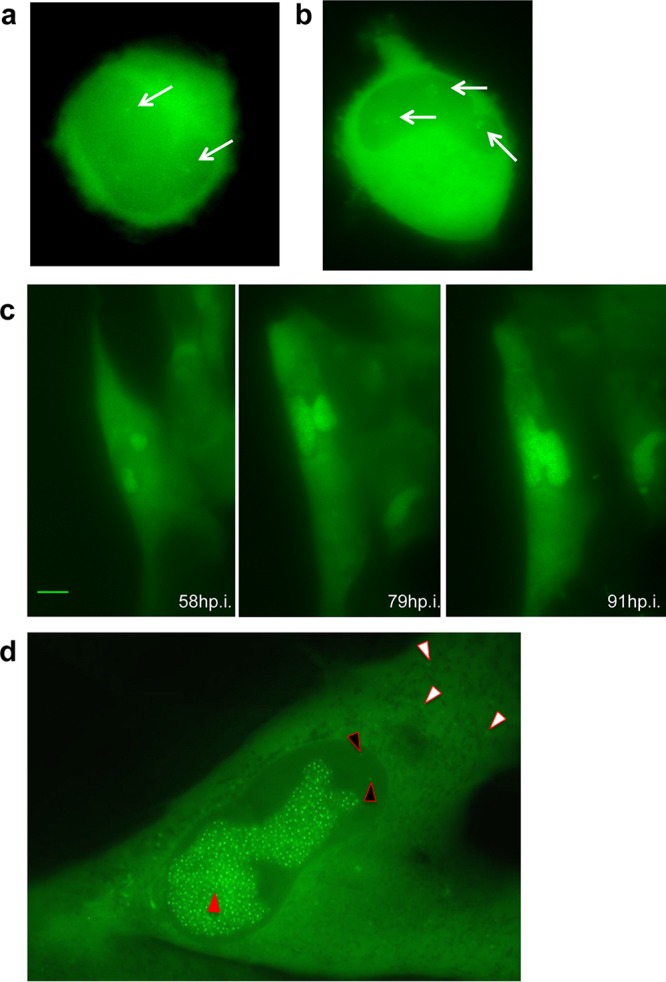
Visualization of ANCHOR-HCMV infection steps in living cells. MRC5 cells were infected with TB40-ANCHOR3 HCMVs at an MOI of 0.5. (a) About 16 or 17 h p.i., a few very faint spots appeared in infected cells, which possibly corresponded to incoming viral genomes (arrows) (magnification, ×63). (b) Distinct areas suggestive of prereplicative sites developed around the initial spots, which multiplied, while these areas increased in size (arrows) (magnification, ×63). (c) Later in infection (around 70 to 80 h p.i.), the areas fused into a unique putative RC, which continued to grow (magnification, ×40; scale bar, 10 μm). (d) An infected MRC5 cell imaged 72 h p.i.; the nucleus contains a large RC with numerous bright spots (red arrowhead), while fainter spots are visible in the nucleus outside the RC (black arrowheads) and in the cytoplasm (white arrowheads) (magnification, ×63). All images were acquired with a wide-field Zeiss Axiovert Observer Z1 with a 1.4-NA 40× or 63× objective.

**FIG 7:**
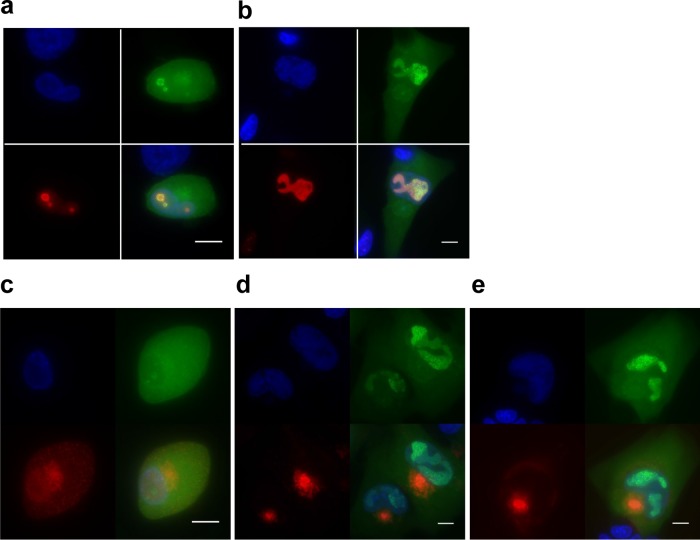
The putative replication compartment stains for the polymerase-associated processivity factor pUL44, while pp28 and gB proteins accumulate in a region close to the nucleus suggestive of the assembly compartment. (a and b) TB40-ANCHOR3 HCMV-infected MRC5 cells were stained for pUL44 at different times p.i. (a) Twenty-four hours p.i. (b) At 72 h p.i., only the putative RC was positive for pUL44, confirming its status. Upper left, Hoechst 33342; upper right, OR-GFP fluorescence; lower left, anti-pUL44; lower right, merge. (c to e) TB40-ANCHOR3 HCMV-infected MRC5 cells were stained for the pp28 tegument protein at different times p.i. (c and d) or for the envelope gB protein (e). (c) Twenty-four hours p.i., pp28 was already expressed but appeared diffuse in the whole cell. (d) Seventy-two hours p.i., pp28 was concentrated in a large region close to the nucleus where OR-GFP spots were also visible, suggesting the region is the assembly compartment. Upper left, Hoechst 33342; upper right, OR-GFP fluorescence; lower left, anti-pp28; lower right, merge. (e) TB40-ANCHOR3 HCMV-infected MRC5 cells were stained for the gB envelope protein 72 h p.i., showing accumulation in a region close to the nucleus, especially in the central area of the region. Upper left, Hoechst 33342; upper right, OR-GFP fluorescence; lower left, anti-gB; lower right, merge. Scale bars, 5 μm.

TB40-ANCHOR3 viruses were used to infect MRC5 fibroblasts for time-lapse imaging of infection progression in live cells. Diffuse GFP fluorescence attributable to the OR3-GFP proteins was first detected in the cytoplasm, as well as in the nuclei, of infected cells between 4 and 5 h p.i. This duration likely corresponds to the time required for the virus to attach and enter a cell, travel to the nucleus, and express its first genes. Interestingly, during the same period, infected cells transiently rounded out before recovering their usual spindle shape (see Fig. S2 in the supplemental material). About 16 h after infection, faint spots were detected in the cells' nuclei (Fig. 6a). The number of spots increased over 2 or 3 days (Fig. 6b), but they remained confined to small specific areas (Fig. 6b), which finally fused (Fig. 6c; see Movie S1 in the supplemental material). About 72 h to 95 h p.i., depending on the cells, a single large and well-demarcated area containing up to several hundred intense spots occupied most of the nuclear space. This nuclear area was highly reminiscent of a replication compartment (RC) previously associated with CMV intranuclear inclusion bodies (26) and later defined as the site of viral DNA replication and replication-specific protein accumulation (27, 28). To better characterize this specialized area, we performed immunofluorescence (IF) staining of ANCHOR3-HCMV-infected cells with anti-UL44 antibodies. The UL44 gene encodes the polymerase-associated processivity factor, a factor specifically recruited to RCs (28). The results presented in Fig. 7 clearly show that this well-demarcated nuclear area containing most of the spots (and the most intense spots) is also precisely costained with the anti-UL44 antibody, indicating it is indeed the RC. Interestingly, UL44 distribution clearly evolved during the course of infection but always coincided with areas where HCMV genomes were also observed. This was true at 24 h p.i., when viral genomes were still moderately amplified and present in limited, rather small areas (Fig. 7a), but also at 72 h p.i., when the different replication zones had fused into a large replication compartment occupying most of the nucleus (Fig. 7b). In addition to the intense spots observed in the RC, numerous fainter spots were clearly visible in the rest of the nucleus and in the cytoplasm (Fig. 6d), where they were especially abundant in a large, demarcated, rounded region adjacent to the nucleus at 72 h p.i. (Fig. 7b). As it is now largely accepted that the viral tegument and envelope are acquired in a specialized cytoplasmic compartment, we tried to better define the zone containing these cytoplasmic spots by IF staining for tegument (pp28) and envelope (gB) viral proteins. In the context of viral infection, pp28 presented a punctate distribution in the whole cell early after infection (Fig. 7c), but after 72 h, little pp28 remained in the nucleus while most of it accumulated in a region adjacent to the nucleus, as described previously (29) (Fig. 7d). Interestingly, at this time, numerous faint spots were also present in the same zone. The same held true for gB staining 72 h p.i., which accumulated in a similar domain where numerous HCMV spots were also clearly visible (Fig. 7e). It is therefore very likely that this structure is the assembly compartment, which overlaps the endoplasmic reticulum-Golgi intermediate compartment (ERGIC), where naked capsids acquire their tegument and envelope (30, 31). Interestingly, the HCMV genomes remained well visible, in addition to the IF-targeted proteins, indicating that enough GFP from the ANCHOR system survived the immunofluorescence procedure, in particular fixation, and that the two approaches are therefore compatible, enabling analysis and colocalization of viruses or viral genomes with cellular or viral proteins of interest.

Following the formation of the large, unique RC, no appreciable change in viral accumulation or cellular morphology seemed to occur for several hours. However, after this apparent quiescence or lag period, membrane rearrangements and cytoplasmic “bubbling” appeared at one or both poles of the cell. These events rapidly amplified and suddenly resulted in cell fragmentation and death, similar to “blebbing” (32), leaving only fluorescent scraps (see Movies S2 and S3 in the supplemental material). It is noteworthy that each cell presented its own infection time course, and some cells underwent a complete cycle from fluorescence appearance to blebbing and fragmentation in less time than the lag period between the mature RC and the cytoplasmic bubbling of others (see Movie S4 in the supplemental material).

### Replicating viral genomes associate with preformed capsids.

**FIG 8:**
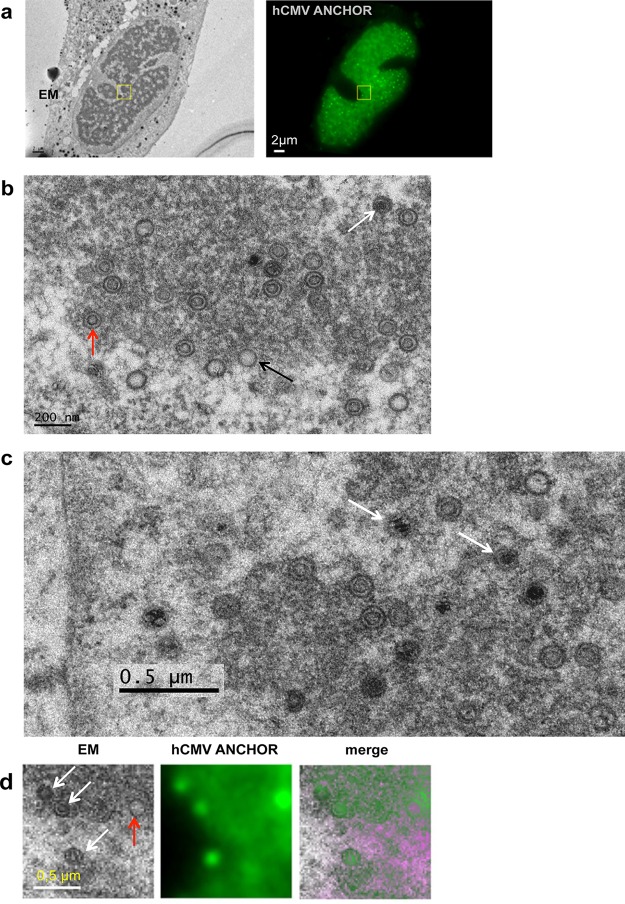
Visualization of infected cell by correlative fluorescence/electron microscopy. (a) Cells infected with TB40-ANCHOR3 HCMV were first analyzed by fluorescence microscopy 96 h p.i. (left) and then fixed and processed for electron microscopy (EM) examination (right). The zone boxed in yellow was further analyzed at higher magnification, as shown in panel d. (b) In the RC, electron microscopy revealed different forms of capsids resembling the typical type A, B, and C capsids (black, red, and white arrows, respectively). (c) At higher magnification, other, possibly more diverse forms of capsids seem to appear, some containing dense fragmented material (arrows). (d) In the chosen area (boxed in yellow in panel a), 4 capsids were observed by EM, all of which correspond to fluorescent spots. Three appeared as B forms (white arrows), while the fourth was an A form (red arrow). Spots associated with the three type B capsids presented similarly weak intensities, suggesting they contained a single viral genome. On the other hand, the type A capsid coincided with a much brighter spot and could be associated with a replicating structure with several genomes.

It is generally accepted that HV replicating genomes associate in the nucleus with preformed capsids whose TER complexes, encoded by the UL89, UL56, and UL51 genes, capture and internalize viral DNA through the portal complex (UL104) (33, 34). We analyzed cells at this stage of infection by correlative fluorescence/electron microscopy (Fig. 8a). Electron microscopy revealed different types of capsids (Fig. 8b), reminiscent of previously described A, B, and C forms (35), but also possibly other forms (Fig. 8c). When merging fluorescence and electron microscopy images, fluorescent spots and viral capsids were nicely superimposed at the periphery of the RC (Fig. 8d). The chosen area (boxed in yellow in Fig. 8a) shows four capsids on the electron micrograph (Fig. 8d), three containing material (possibly type B) and one that appears empty. Interestingly, the fluorescence staining type B capsids (which are at the edge of the RC) was weaker than that associated with the other capsid but equivalent between type B capsids, suggesting that these capsids already contain a single viral genome. On the other hand, the empty capsid could be linked to a replicative structure containing more than one viral genome.

### The ANCHOR technology enables quantitative tracking of HCMV infection.

**FIG 9:**
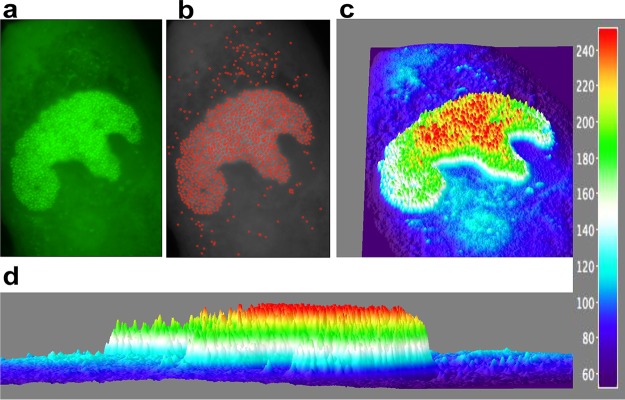
ANCHOR technology allows quantification of HCMV infection in live cells. (a) Fluorescent spots in an MRC5 cell infected with TB40-ANCHOR3 HCMV 72 h p.i. (b) Same as panel a but treated to filter out the fluorescent background and then processed using the spot detector plug in for ImageJ (spot radius, 2; cutoff, 0; percentile, 7). (c and d) The images were then converted into a 3D intensity surface plot (perspective) (c) or a picture in *x* and *z* (d) to assess particle intensities. A single viral genome corresponds to 30 FU. In the RC, all the spots harbored between 2 and 5 viral genomes, and this number decreased from the center to the periphery, suggesting a highly organized territory. Outside the RC, only unique genomes were observed (see the text for an explanation).

The number of ANCHOR spots present in a particular cell can be determined using the Image J particle detector software (particle detector and tracker). In the cell illustrated in Fig. 9, we detected 1,155 ANCHOR foci distributed among the RC (*n* = 1,005), the remaining non-RC nucleoplasm (*n* = 16), and the cytoplasm (*n* = 134). The fluorescence intensities of the observed spots were highly variable (Fig. 9a and b) and could be quantified (Fig. 9c) using an approach similar to the one that enabled precise quantification of Escherichia coli replisomes and yeast telomerase (36). Briefly, we used the three-dimensional (3D) interactive surface plot plug in for ImageJ, which converts fluorescence intensity into arbitrary fluorescence units (FU) on the *z* axis of the 3D reconstruction. Therefore, foci are not represented as 2D dots but as 3D peaks, where the *z* values correspond to fluorescence intensities. The diffuse background GFP fluorescence in the cytoplasm and the nucleus (outside the RC) appears dark blue in Fig. 9c and may be assigned a value of 90 arbitrary units (AU) on the color scale of Fig. 9. In the same areas, pale-blue regions and spots corresponding to ±120 AU are also visible, with some of the peaks superimposed on the spots present on Fig. 9a and b. Interestingly, the RC itself is delimited by a line of spots of the same color. Inside the RC, colors are not distributed along a uniform gradient but rather in well-defined successive concentric zones whose mean intensities radially decrease from the center (containing 240- and 210-AU spots) to the periphery and which are separated by ±30 AU (Fig. 9c and d). A similar distribution was also observed in ARPE-19 cells (see Fig. S3 in the supplemental material), suggesting it results from a phenomenon common to all infected cells.

### ANCHOR-HCMV is a new tool for rapid and cost-effective assessment of antiviral compounds.

**FIG 10:**
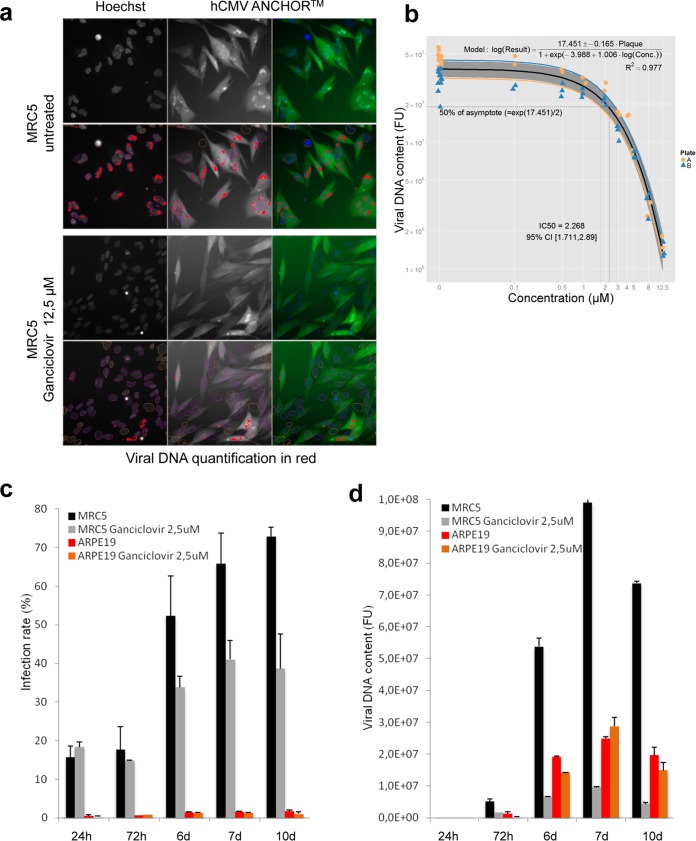
Cell-type-specific effects of ganciclovir on TB40-ANCHOR3 HCMV infection and IC_50_ determination by automated high-content imaging. (a) TB40-ANCHOR3 HCMV-infected cells were treated or not with 12.5 μM ganciclovir; 72 h p.i., the cells were observed using a Thermo Scientific Cellomics Arrayscan Vti microscope, and the images were analyzed with the compartmental analysis algorithm, allowing detection of viral DNA (red) (see Materials and Methods for an explanation). (b) Experiment similar to that shown in panel a, but TB40-ANCHOR3 HCMV-infected cells were treated with increasing doses of ganciclovir. The results of the quantification were plotted against ganciclovir concentrations (see the text), allowing precise determination of the IC_50_ and IC_90_. Two identical experiments were performed on separate plates (A and B). (c) Time course of TB40-ANCHOR3 HCMV infections in MRC5 or ARPE-19 cells in the presence or absence of 2.5 μM ganciclovir. In MRC5 cells, infection progressed to more than 70% infected cells 10 days p.i., and the infection was partly controlled by ganciclovir. ARPE-19 cells did not seem to be highly permissive, and only 2 to 3% of the cells became infected in the presence or absence of ganciclovir. (d) Same as panel c but using viral DNA quantification as a readout. d, days. The error bars represent SD.

To date, quantitative information about the presence of HCMV or its replication in a sample mainly relies on quantitative-PCR (qPCR)-based technologies which, despite being very tedious, remain indispensable from a clinical point of view (37). For this kind of investigation and biotechnological applications, the ANCHOR technology also represents a very promising alternative. We analyzed the infection kinetics of MRC5 cells in the presence or absence of ganciclovir, a compound widely used to treat HCMV infection (38–40). We have developed an Arrayscan-based custom algorithm for automated image analysis. To quantify the viral DNA content of cells infected with our TB40-ANCHOR3 HCMV viruses, this algorithm is remarkably efficient and enables direct determination of infection and replication rates per cell and/or per population (Fig. 10a). Imaging of ganciclovir-treated cells revealed drastically reduced viral DNA content. Despite the initial appearance of fluorescent particles in discrete nuclear domains, subsequent massive amplification and RC formation were inhibited (see Fig. S4 in the supplemental material). Using various concentrations of ganciclovir, the 50% inhibitory concentration (IC_50_) was determined to be 2.26 μM, a value within the range measured by other techniques, while the IC_90_ was measured at 8.435 μM (41). When two independent plates were analyzed, intra- and interplate variability was remarkably low, with a correlation coefficient of 0.97 (Fig. 10b), demonstrating that our experimental approach is highly reproducible and robust. We next tested infectivity and response to drug treatment in parallel in two different cell lines; in a pilot experiment, we found that at an MOI of 0.5, the infection rate of MRC5 cells increased from 15 to 75% between 24 h and 10 days p.i. In the presence of 2.5 μM ganciclovir, this increase was limited to 40% between days 7 and 10, with no effect at 24 h p.i., as expected for a drug blocking the viral polymerase and not virus entry. In contrast, the infection rate of ARPE-19 cells infected at an MOI of 0.5 remained constant between 1 and 3% during the entire experiment, with no evident effect of ganciclovir (Fig. 10c). Viral DNA content was reduced by 95 to 100% in ganciclovir-treated MRC5 cells 7 or 10 days p.i., while surprisingly, in ARPE-19 cells, the viral DNA content increased until day 7 p.i. Despite a slight decrease at day 10 p.i., no effect of ganciclovir was observed at 2.5 μM (Fig. 10d) in these cells, which required 12.5 μM to completely abolish HCMV replication, indicating that sensitivity to ganciclovir is cell type dependent (data not shown). The ease of this quantification paves the way for innovative screening strategies in the search for new antiviral drugs.

## DISCUSSION

In this study, we describe the first application of the ANCHOR DNA labeling technology to a virus, resulting in ANCHOR-modified HCMVs that allowed follow-up and quantification of HCMV infection and replication kinetics in real time and living cells from a few hours p.i. until lysis of the infected cell. The biologies of ANCHOR-HCMVs and parent BAC-derived viruses appear similar, with the replication rate of TB40-ANCHOR3 being even more robust (Table 2 and Fig. 3). This increased replication rate may stem from variations in virus stock titration, but we may also have selected a virus particularly apt to thrive in MRC5 cells during the BAC construction. However, this TB40-ANCHOR3 virus behaves as expected and seems, therefore, to be a representative HCMV with no significant compensatory deletion, like those that have been reported to sometimes affect HCMV-BAC over long genomes (42). Moreover, TB40-ANCHOR3 viruses are tegumented and enveloped and contain viral DNA, possibly associated with OR-GFP proteins (Fig. 4). It may seem surprising that a number of OR-FP proteins sufficient to allow detection of a fluorescent spot enter the capsid, because the viral genome is usually supposed to occupy the entire inner volume of the capsid (43–45). This number has been estimated to be around 50 (46), while the number of OR-GFP proteins associated with a single ANCH site was evaluated as ±500 by FCS on a chromosomal site integrated into the genome of a human cell (23). Using 1.15 nm (half the distance between concentric layers of packed DNA [45]) and 0.85 × 10^5^ nm as the radius and length, respectively, of a 250-kb viral DNA, the calculated volume of the viral DNA (3.53 × 10^5^ nm^3^) is less than the volume of a capsid with an internal radius of 48 to 50 nm (43) (4.63 × 10^5^ to 5.25 × 10^5^ nm^3^), and the remaining volume could easily accommodate up to 500 OR-FP proteins that would occupy only an estimated volume of 5.25 × 10^4^ nm^3^. Therefore, volume considerations do not preclude the presence of OR-GFP proteins inside the capsid even if the mechanism governing their introduction into the capsid remains unknown. It is notable that OR-GFP proteins cannot enter any kind of viral particle (results not shown; 60), indicating that each viral family has its own packaging specificities. ANCHOR-HCMV genomes are remarkably stable, because >90% of the viruses conserve the ANCHOR phenotype through a 10^9^ amplification step. Similar results were also obtained with TB40-GFP, suggesting that the presence of the ANCHOR components does not change stability. It was firmly established in yeast, Drosophila, and human cells that OR-FP proteins bind to their specific target sequences and form fluorescent spots that are easily visualized by fluorescence microscopy (21, 22, 47). In this paper, we demonstrate that this is also true in ANCHOR-HCMV-infected cells. While ChIP experiments have shown that OR-GFP proteins specifically bind to the ANCH sequence of the viral genome, spots are observed only when cells infected with an ANCHOR-HCMV also express the corresponding OR-FP protein. The same OR-FP protein in the presence of a non-ANCHOR virus genome does not form any spots. Therefore, the spots we observed in this study each represent a cluster of OR-FP proteins specifically complexed to ANCH (and surrounding) sequences inserted in viral genomes, whether unique or in a concatemeric form, nude or encapsidated.

The first sign of infection of MRC5 cells by ANCHOR-HCMV was the appearance of diffuse fluorescence in the entire cell about 4 to 5 h p.i., attributable to OR-GFP expression. This fluorescence increased gradually, mainly in the cytoplasm, until about 16 h p.i., when one or a few discrete spots, likely corresponding to the original incoming viral genomes, became visible in the nucleus. From that moment, the complete course of infection could be observed. These initial spots first multiplied in small specific territories within the nucleus called prereplicative sites. As this first modest multiplication occurred rather early during infection, it is not clear whether the viral polymerase was already expressed and active at this stage or whether the amplification was performed by cellular polymerases. In herpes simplex virus 1 (HSV-1)-infected cells, inhibitors of viral DNA replication block the formation of RCs, but not of similar prereplicative sites (48, 49). Interestingly, this also seems to hold true for HCMV, as TB40-ANCHOR3-infected MRC5 cells present only prereplicative structures, but not mature RCs, when treated with ganciclovir (see Fig. S4 in the supplemental material). This initial prereplicative stage was followed by a massive amplification step, resulting in numerous (several hundred) very intense spots that, after fusion of the different viral replication domains, clustered in a unique large nuclear compartment at about 72 h p.i. At this stage, only this compartment was precisely and uniformly stained with an anti-UL44 antibody and thus contained the polymerase-associated processivity factor characteristic of the RC. Interestingly, ANCHOR technology enables quantification of the fluorescence intensity of single spots and discrimination between background and viral-genome-associated fluorescence. Spot intensity was highly variable, with the brightest spots in the RC while those found outside the RC or in the cytoplasm seemed similarly weak. These scattered spots (Fig. 9, pale blue), corresponding to ±120 AU, were visible over the 90-AU intensity of the diffuse GFP fluorescence background (dark blue) grossly corresponding to the cytoplasm of the cell (Fig. 9). The RC was also delimited by a line of spots of similar intensity. In the RC itself, fluorescence values did not form a continuous gradient but varied in discrete steps, separated by ±30 AU. As each spot corresponds to one or more viral genomes and each viral genome contains a single ANCH target sequence that binds and recruits a similar number of OR-FP proteins, we assume that the stepwise variation of the fluorescence intensity correlates with the number of viral genomes present in each spot. Because spot intensities in the RC varied by steps of 30 AU, which is also the difference between the background and the less intense spots, it seems logical to speculate that 30 AU is the quantity of fluorescence generated by the OR-GFP proteins associated with a single viral genome. Therefore, spots of 120, 150, 180, 210, and 240 AU could correspond to 1, 2, 3, 4, and 5 viral genomes after correcting for the 90-AU fluorescence background. In the RC, multiple viral genomes could correspond to concatemers generated by a rolling-circle replication mechanism that would subsequently be cut by the terminase complex after encapsidation of a single genome unit (50). This rolling-circle mechanism of lytic replication, similar to phage replication and widely accepted, has never been formally proven but fits very nicely with our data, even if we cannot exclude other mechanisms involving θ structures, for instance (1, 51). If this assumption is correct, low-intensity RC and, non-RC nuclear and cytoplasmic particles are probably capsids containing a single viral genome but at different stages of maturation, while bright spots present in the RC and displaying between 150 and 240 arbitrary FU are replicative structures harboring between 2 and 5 viral genomes. The most intense spots are preferentially localized in the center of the RC, and the fluorescence decreases toward the edge in concentric zones with discrete values (Fig. 9c and d). A similar distribution of spot intensities was also observed in ARPE19 cells (see Fig. S3 in the supplemental material), suggesting that the RC is highly organized, with active replication occurring in the center of the RC, and that this organization is a general feature of HCMV replication in infected cells. The concatemeric structures preferentially produced in the center of the RC could migrate toward the periphery and would be progressively shortened as they encountered preformed empty capsids, out of which the terminase complex cuts unit-long complete genomes, which are encapsidated. Figure 9c suggests that this migration/encapsidation process arrives at completion at the nuclear membrane, which is underlined by a brim of single viral genome spots. Finally, considering that most of the spots are in the RC and associated with 1 to 5 genomes, one can assume that such an infected cell contains at this precise moment 3,000 to 5,000 viral genomes. It is noteworthy that qPCR titration of viral genomes yields values ranging from 8,000 to 20,000 viral genomes per infected cell (Table 2). We consider these two sets of values based on spot counting and fluorescence intensity remarkably coherent, especially as they correspond to instantaneous (fluorescence) and cumulative (qPCR) measurements.

Once synthesized, the viral genomes have to enter capsids that are assembled in the nucleus from the different capsid proteins imported from the cytoplasm. With their portal complex, capsids are able to cleave genome concatemers resulting from the rolling-circle mechanism and to internalize a single unit-length genome through an ATP-dependent mechanism (33). When the RC was analyzed by correlation fluorescence/electron microscopy, unexpected results were obtained, suggesting that the classical capsid classification into A, B, and C forms is possibly oversimplified. From analyzing numerous images (Fig. 8 and results not shown), it appeared that capsids that could be classified as C forms are rare and much less frequent than reported (35). On the other hand, some capsids containing dense fragmented material are also clearly visible in Fig. 8c. Of course, all these results could simply reflect the different conditions of infection and sample treatment, but could also indicate that more capsid forms exist that need to be understood. Whatever their significance, the three capsids on the left in Fig. 8d (marked with white arrows) were associated with three spots of equal intensity, but less intense than the spot on the right in the same image. This association could, of course, be fortuitous, but it is also tempting to speculate that these three spots represent capsids that have internalized a single viral genome and that are ready to leave the RC. The fourth capsid, which appeared empty in electron microscopy, is in close contact with a stronger fluorescent signal and could thus be associated with a replicating concatemeric structure. However, the three capsids showing weak fluorescence resemble type B capsids, considered to be devoid of genetic material. This interpretation, therefore, remains highly speculative, but on the other hand, simple random association is also hard to imagine. Hence, it is possible that other intermediate forms of capsids exist and that type B is a heterogeneous population, a fraction of which could contain DNA.

Once capsids have loaded a viral genome, they leave the RC and the nucleus for the cytoplasm. ANCHOR technology enables precise counts of the genomes present in different parts of the cell, as illustrated in Fig. 9. Due to their fluorescence values, we postulate that spots present in the nucleus outside the RC and in the cytoplasm correspond to single encapsidated genomes, in contrast to spots observed in the RC. If this interpretation is correct, significantly fewer viral genomes would be in the non-RC nucleoplasm and the cytoplasm than in the RC, possibly around 1% or less. Interestingly, when titers of virus stocks are plotted against the number of producing cells, the numbers obtained are closer to what is found in the cytoplasm than to what is found in the RC, suggesting that most of the genomes synthesized in the RC could be lost. Therefore, the passage from the RC toward the cytoplasm could be a significant bottleneck in virus production even if the very low number of spots observed in the non-RC nucleoplasm could argue for the RC exit being the true limiting step. It is also possible that once encapsidated with the viral genome, the fluorescence intensity of OR-FP is reduced, and that only a minute fraction of the mature viruses remain visible. Reduced fluorescence of the encapsidated viral genome could be due to loss of OR-FP molecules during the encapsidation process. The intensity of cytoplasmic spots seen in Fig. 9a and b, which does not reach the threshold of 120 fluorescence AU, is consistent with this hypothesis. Indeed, a cluster of 50 to 100 OR-FP proteins is already detectable, and it is therefore logical to find in the cytoplasm spots with intensities ranging between background and 120 AU. This may also explain why we have so far been unable to visualize incoming viruses during the very first hours p.i. although they could simply have been missed due to the extremely low probability of detecting them in an adequate focal plane. A large proportion of the spots found in the cytoplasm preferentially clustered in a faintly demarcated region close to the nucleus. Because this region is stained by anti-pp28 and anti-gB antibodies (Fig. 7), it very likely represents a viral assembly compartment in which the capsids acquire their tegument and envelope and which overlaps the ERGIC (29–31). In the rest of the cytoplasm, spots were fainter, and their observation necessitated boosting the image acquisition conditions, leading to RC signal saturation. Time-lapse imaging of infected cells also revealed sudden blebbing and fragmentation of infected cells. This blebbing is often considered indicative of apoptosis (32), but the mechanism of fragmentation ending cell infection is poorly understood. Because the timing of this fragmentation is significantly delayed by the potent suppressor of apoptosis vMIA encoded by the UL37x1 viral gene, it is likely that a longer infection time is advantageous for the virus and that fragmentation, on the other hand, interrupts the viral cycle and generates alarm signals to neighboring cells (52). Interestingly, this ultimate step occurred at very different times in different cells and produced numerous highly fluorescent cell fragments possibly containing viral DNA. It will be of great interest to further explore this process to determine whether it may contribute to virus dissemination or whether it is the ultimate cellular defense, bypassing the final maturation steps of the viruses and releasing noninfectious but immunogenic material.

From a biotechnological point of view, ANCHOR-HCMVs are a remarkable tool to characterize antiviral compounds. As a proof of concept, we measured the effect of ganciclovir on the infection of various cell lines with ANCHOR3-HCMV. With very limited hands-on time investment, we were able to establish the IC_50_ and IC_90_ of the drug for the HCMV infection of MRC5 human fibroblasts, simply recording fluorescence variation using an automated Arrayscan microscope (Fig. 10). The results were highly reproducible and consistent with values previously published (41). We performed the same experiment on the retina epithelial ARPE-19 cell line, with totally different results. These cells were indeed much less efficiently infected by the ANCHOR-HCMV TB40, and the course of infection did not seem to be modified by ganciclovir, suggesting that the drug is not metabolized in the same manner in MRC5 and ARPE-19 cells. In the latter, higher drug concentrations may be needed, as suggested by the absence of HCMV replication in these cells in the presence of 12.5 μM ganciclovir. Whatever the reasons underlying the different responses of different cell lines to ganciclovir, these results indicate that ANCHOR-HCMVs will permit rapid and cost-effective screening of large libraries of chemicals in search of new antiviral activities, including measurements of such parameters as the toxicity of the compound, infection rate, virus DNA replication level, and infection propagation without any fixation, extraction, or reagent. Using ANCHOR-HCMV and automated high-content microscopy, it will be easy to screen even large collections of chemical compounds. Furthermore, the technology can be used to label other DNA viruses for which we already have proofs of concept (results not shown).

In addition to new insights into the fundamental biology of numerous DNA viruses, ANCHOR technology is amenable to high-throughput imaging, with high confidence over long time series of multiple cell lines under different biological conditions in parallel. The technology is therefore particularly suited to rapidly testing compound concentrations, stability, and administration conditions for the design of new and/or combinatorial antiviral treatments. For all these reasons, the ANCHOR technology appears to be a highly promising tool for fundamental research, but also for numerous biotechnology applications.

## MATERIALS AND METHODS

### Viruses and BACs.

The TB40/E HCMV strain was obtained from a throat wash of a bone marrow transplant recipient patient (53), and its genome was cloned as a BAC in E. coli by replacing the nonessential US2 to US7 viral genes with the BAC vector pEB1097 (53, 54). This construct was later modified by inserting the GFP gene under the control of the mCMV-MIEP in the vector sequence, producing the TB40-GFP BAC (E. Borst, personal communication). This BAC has been maintained, amplified, and mutated in E. coli DH10B bacteria grown in LB broth supplemented with the appropriate antibiotics. For the production of viruses from BACs, BAC DNA was first purified from bacteria using a PureLink HiPure plasmid DNA purification kit (Invitrogen) or a NucleoBond Xtra BAC kit (Macherey-Nagel) according to the manufacturer's specific instructions. DNA was then transfected in MRC5 permissive human lung fibroblasts with X-tremeGene HP or X-tremeGene 9 transfection reagents (Roche) following the provided recommendations. When cytopathic effect reached nearly 100% of the cells, the content of the flask was harvested and centrifuged for 10 min at 2,000 rpm to remove cell debris, and the supernatant was centrifuged at 25,000 rpm (106,000 × *g*) for 45 min at 16°C in a SW32Ti rotor (Beckman) on a 20% sucrose cushion. Alternatively, after the first centrifugation, the supernatant could also be centrifuged at 20,000 rpm (48,500 × *g*) for 90 min at 16°C in a JA25.50 fixed-angle rotor on a 20% sucrose cushion with a similar virus yield. With both techniques, easily visible pellets were obtained under the cushion, resuspended in Dulbecco's modified Eagle's medium (DMEM)-20% fetal calf serum (FCS), aliquoted in vials, and frozen at −80°C. The ANCHOR-modified HCMV BACs were derived from the TB40-GFP BAC (a kind gift from E. Borst and M. Messerle). As a first proof of concept, the TB40-GFP BAC was initially modified by introducing an ANCH2 target sequence instead of the mCMV-MIEP-GFP gene. Briefly, the ANCH2 sequence and a kanamycin resistance gene were amplified, respectively, from the pUC18-ANCH2 (22) and the pORI6K-5FRT (a kind gift from M. Messerle) plasmids using PrimeStar Max 2X (TaKaRa) according to the manufacturer's recommendations. The fragments were then purified, phosphorylated, and ligated, and the ligation product was used as the template for a second amplification between new primers, selecting the required product of ligation and introducing at both extremities 50-bp homology sequences (H1 and H2) necessary for the final recombination of the product in the TB40-GFP BAC. This H1-Kana^r^-ANCH2-H2 cassette (Fig. 2) was cloned between the PvuII sites of the pGEM-7Zf(+) vector (Promega), using NdeI or ApaI linkers (pGΔANCH2-kana). DH10B bacteria containing the TB40-GFP BAC were first transformed with the pKD46 vector encoding the arabinose-inducible phage Red α, β and γ recombinases, and then, with the purified H1-Kana^r^-ANCH2-H2 cassette. Recombinant clones were obtained and analyzed by BamHI digestion profile, and the clone TB40-ANCH2-Kana was finally verified by sequencing (Fig. 2). This BAC was amplified, purified, and transfected into MRC5 human fibroblasts. Complete cytopathic effects were observed 4 weeks later, and at that time, the content of the flask was harvested and viruses were purified as described above. We next created the TB40-ANCHOR3 BAC by replacing the mCMV-MIEP-GFP sequence of the TB40-GFP BAC with a cassette containing the ANCH3 target sequence, a chimeric gene encoding the OR3 protein fused to GFP under the control of an SV40 promoter, and a kanamycin resistance gene as a selection marker. This cassette was derived from the previously obtained pGΔANCH2-kana plasmid, whose ANCH2 sequence had been removed by PmlI digestion and replaced with an ANCH3 sequence, producing construct pGΔANCH3-kana. The OR3 gene had already been cloned into the peGFPc1 vector (Clontech), and an SV40 promoter was inserted directly upstream of the GFP-OR3 gene, producing the pSVGO3 plasmid. The pSV40GFP-OR3 cassette was excised from the pSVGO3 plasmid and inserted into the MluI/PvuII sites of the pGΔANCH3-kana plasmid, creating the pG7ΔKanaSVOR3GFPANCH3 vector. Finally, the cassette of interest with the structure H1-kana-OR3GFP-pSV40-ANCH3-H2 was excised by NdeI digestion, agarose gel purified, and used for recombination in TB40-GFP containing DH10B bacteria. The clones obtained were screened by BamHI, EcoRI, or HindIII restriction profile, and one of them, showing the expected modification, was named TB40-ANCHOR3 and further confirmed by PCR and DNA sequencing (Fig. 2). This clone was then amplified, purified, and transfected into MRC5 human fibroblasts. Complete cytopathic effects were observed 5 weeks later. At this time, the viruses were purified and stored as described above. To create a pure ANCHOR-modified viral stock from this first production, 10^4^ MRC5 cells plated in 96-well plates (imaging grade; Corning Cell Bind) were infected with 0.5 PFU per well. Less than half of the wells displayed infection, and one isolated green-fluorescent plaque could be recovered and used to infect 10^4^ fresh MRC5 cells. After cell lysis, the culture medium was transferred to two T175 flasks of MRC5 cells, which were incubated until cytopathic effects were estimated to be maximal (12 weeks from plaque picking). At this time, the content of the flasks was harvested, and viruses were purified as described above, providing a stock of 8 × 10^8^ TB40-ANCHOR3 PFU in total.

### Plaque-forming assay.

For quantification by plaque-forming assay, 10^5^ MRC5 cells/well were plated in 24-well plates. The next day, the culture medium was removed, and the cells were infected with various dilutions of the virus stock to be titrated. After 2 or 16 h contact, virus dilutions were removed and replaced with fresh supplemented culture medium containing 0.5% low-melting-point agarose, and plaques were allowed to develop until they became visible. At this time, the cells were fixed by addition of 1 ml of 2% formaldehyde, and plaques were counted after staining with 0.02% methylene blue (55).

### Replication rate assay.

To measure replication rates, 10^5^ MRC5 cells/well were seeded in two 24-well plates and infected with the TB40-GFP or the TB40-ANCHOR3 virus at an MOI of 0.2. After 3 h of contact, the supernatants were removed and replaced with fresh medium, and the plates were incubated at 37°C under 5% CO_2_. One of the plates, with a glass bottom, was used to determine the numbers of cells and of fluorescent infected cells at each time point. Supernatants and cells were separately collected from duplicate wells of the second plate 1, 2, 4, 6, 8, or 10 days postinfection. The supernatants were directly extracted with a phenol-chloroform-isoamyl alcohol mixture and ethanol precipitated in the presence of HCMV-free carrier DNA. After centrifugation, the DNA pellets were resuspended in 100 μl sterile Tris-EDTA (TE) and conserved at 4°C. DNAs from the infected cells were purified using a NucleoSpin DNA RapidLyse kit (Macherey-Nagel; reference no. 740100.50) according to the manufacturer's instructions and eluted in a final volume of 200 μl. Viral DNA quantification was performed in triplicate by qPCR using a LightCycler 480 SYBR Green I master kit (Roche; 04707516001). The cloned UL79 gene was chosen as the reference, and the fragment flanked by primers UL79.1F (CAGATTAGCGAGAAGATGTCG) and UL79.1R (CAGGTTGTTCATGGTTTCGC) was amplified with a PCR efficiency ranging between 1.999 and 2.007.

### Cells, culture, and media.

MRC5 (CCL-171) human primary fibroblasts and ARPE-19 (CRL-2302) human retinal pigmented epithelial cells were obtained from ATCC and grown in DMEM without phenol red (Gibco) supplemented with 10% fetal bovine serum (FBS), 1× penicillin-streptomycin, 1 mM sodium pyruvate, and 1× Glutamax (Gibco). HUVEC, a kind gift from Melinda Benard, were grown in EGM-2 medium, including supplement (PromoCell).

### ChIP.

ChIP assays were performed as described by Metivier et al. (56) with minor modifications. MRC5 cells infected at an MOI of 0.5 with TB40-GFP or TB40-ANCHOR3 virus were treated 72 h p.i. with 1.5% formaldehyde for 10 min. Cross-linking was stopped with 1 M glycine for 30 s, and the cells were washed with cold phosphate-buffered saline (PBS). After preparation of the nucleus with buffer NCPI (10 mM EDTA, 0.5 mM EGTA, 10 mM HEPES, and 0.2% Triton X-100) and buffer NCPII (1 mM EDTA, 0.5 mM EGTA, 10 mM HEPES, and 200 mM NaCl), cell lysis was performed (10 mM EDTA, 50 mM Tris-HCl [pH 8.0], 1% SDS, 1× protease inhibitor cocktail [Roche Biochemicals, Mannheim, Germany]). Subsequently, chromatin was either digested for 3 h at 37°C with DrdI and ScaI restriction enzymes and sonicated three times for 10 s each time or not treated at all. Immunoprecipitations were performed overnight in the presence or absence of 2 μg of selected antibody. Complexes were recovered by a 2-h incubation with protein A Sepharose CL4B saturated with salmon sperm DNA. Beads were sequentially washed in buffer I (2 mM EDTA, 20 mM Tris-HCl, pH 8.1, and 150 mM NaCl), buffer II (2 mM EDTA, 20 mM Tris-HCl, pH 8.1, 0.1% SDS, 1% Triton X-100, and 500 mM NaCl), and buffer III (1 mM EDTA, 10 mM Tris-HCl, pH 8.1, 1% Nonidet P-40, 1% deoxycholate, and 250 mM LiCl) and three times with Tris-EDTA buffer. The washed resin was resuspended in elution buffer (1% SDS, 0.1 M NaHCO_3_) with 30-min incubation, and the cross-link was reversed at 65°C overnight. DNA was purified with QIAquick columns (Qiagen, France). After immunoprecipitation with anti-GFP antibodies, PCR was performed with the following oligonucleotides: ANCH3-P7 (sense [S]) and (antisense [AS]) (NeoVirTech), CMV-P3 (S) CCGTACTTCGTCTGTCGTTT and (AS) TGTGTCTGTTTGATTCCCCG, CMV-P1 (S) ACGGCAAGTCCATAATCACC and (AS) GACCGATCCCACCAATTCTC, and GFP-P1 (S) ACGTTGTGGCTGTTGTAGTT and (AS) GACTTCTTCAAGTCCGCCAT.

### High-content imaging.

MRC5 cells were seeded at 10^4^ cells/well in Corning Cellbind black glass-bottom 96-well plates and infected 24 h postseeding with ANCHOR engineered HCMV at various MOI. For analysis, cells were directly stained with Hoechst 33342 (1 μg/ml) and imaged using a Thermo Scientific Cellomics Arrayscan Vti microscope. Compartmental analysis was used to detect and quantify the infection rate, i.e., the number of GFP-positive cells versus the total number of cells. For spot counting and measurement of viral DNA accumulation in the nuclei of infected cells, we used a spot detector plug in for ImageJ with the following settings: spot radius, 2; cutoff, 0; percentile, 7. Measurements were done for 1,000 cells, and averages and standard deviations (SD) from triplicate experiments are shown. Full analysis protocols for Arrayscan imaging are available upon request.

### IC_50_ calculation.

In order to determine the IC_50_, nonlinear regression was applied, using the natural logarithm of the viral DNA content as a dependent variable, the natural logarithm of the concentration as a continuous predictor, and the plate as a covariate according to the following model: ln(viral DNA content [FU]) = (μ ± γ × plate)/{1 + exp[α + β × ln(concentration [μM])]}, where α, β, γ, and μ are the parameters of a nonlinear regression. The nonlinear regression was realized using R, and the results are presented using the ggplot2 package (57).

### Fluorescence imaging.

Live microscopy was performed using a Zeiss Axio Observer Z1 and an Apotome 2 wide-field fluorescence microscope. The conditions of acquisition are detailed in the figure legends.

### Immunofluorescence.

For immunofluorescence analysis, TB40-ANCHOR3-infected MRC5 cells were fixed in PBS containing 3.6% formaldehyde at different times p.i., washed, and then permeabilized in 10 mM HEPES containing 0.5% Triton X-100 and 1% bovine serum albumin (BSA). After washing, the cells were incubated for 1 h at room temperature with the first antibodies diluted according to the manufacturer's recommendations, washed again, and further incubated for 45 min in appropriately diluted secondary antibodies. The cells were washed again and stained with Hoechst 33342 at a final concentration of 1 μg/ml before fluorescence microscopic examination. For direct immunofluorescence of viral particles, diluted viruses were spotted on poly-l-lysine-precoated Ibidi glass bottom 35-mm dishes. Following a 10-min incubation, immunofluorescence was performed as described above, with slight modifications. Due to the small size of viral particles, incubations were shortened to 30 min for primary antibodies and 15 min for secondary antibodies.

### Correlative light and electron microscopy.

MRC5 cells were grown on MatTek dishes with a finder grid (58) and infected with the TB40-ANCHOR3 virus at an MOI of 1. Four days postinfection, the cells were fixed with 0.05% glutaraldehyde (GA) and 4% paraformaldehyde (PFA) for 30 min at room temperature. Image acquisition and analysis were performed on an Olympus IX81 epifluorescence microscope equipped with a 100× objective lens (UPlan SApo 1.4 oil), a SpectraX illumination system (Lumencore), and a CMOS (complementary metal oxide semiconductor) camera (Hamamatsu Orca-Flash 4.0). Stacks of 51 images each, with a step size of 0.1 μm, were taken. The cells were then fixed overnight with 2.5% GA in cacodylate buffer, pH 7.2, and postfixed in 1% osmium in distilled water for 30 min. The samples were then rinsed in water, dehydrated in an ethanol series, and embedded in Epon. Sections were cut on a Leica Ultracut microtome, and ultrathin sections were mounted on Formvar-coated slot copper grids. Finally, thin sections were stained with 1% uranyl acetate and lead citrate and examined with a transmission electron microscope (Jeol JEM-1400) at 80 kV. Images were acquired using a digital camera (Gatan Orius) at ×200 and ×2,500 magnification. Alignments were performed as described previously (59).

## Supplementary Material

Supplemental file 1

Supplemental file 2

Supplemental file 3

Supplemental file 4

Supplemental file 5
