# Supplementary material for: Real-Time Visualization and Quantification of Human Cytomegalovirus Replication in Living Cells Using the ANCHOR DNA Labeling Technology
Source: J Virol. 2018 Aug 29;92(18):e00571-18. doi: 10.1128/JVI.00571-18 (PMC6146708; doi:10.1128/JVI.00571-18)
Supplement: Supplemental file 1 [file zjv017183805s1.pdf]

Fig.S1

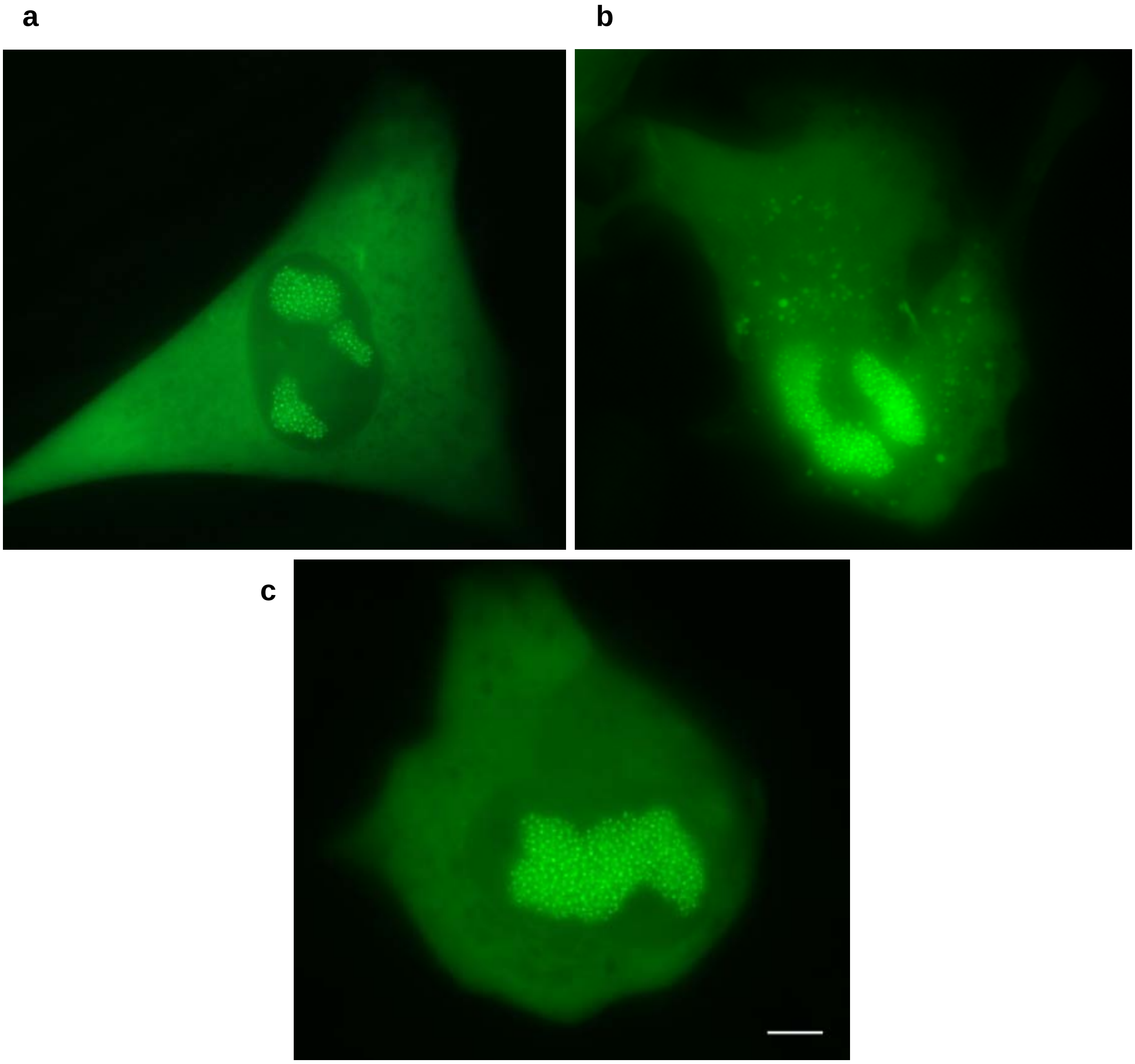

Figure S1. TB40-ANCHOR3 HCMV has retained the cellular tropism of the parental TB40 strain. a)TB40-ANCHOR3 HCMV infection of MRC5 cells, 48h pi.; b) TB40-ANCHOR3 HCMV **i**nfection of ARPE-19cells, 120h pi.; c) TB40-ANCHOR3 HCMV **i**nfection of HUVEC cells, 120h pi.. Barr scale 5µm.

Fig.S2

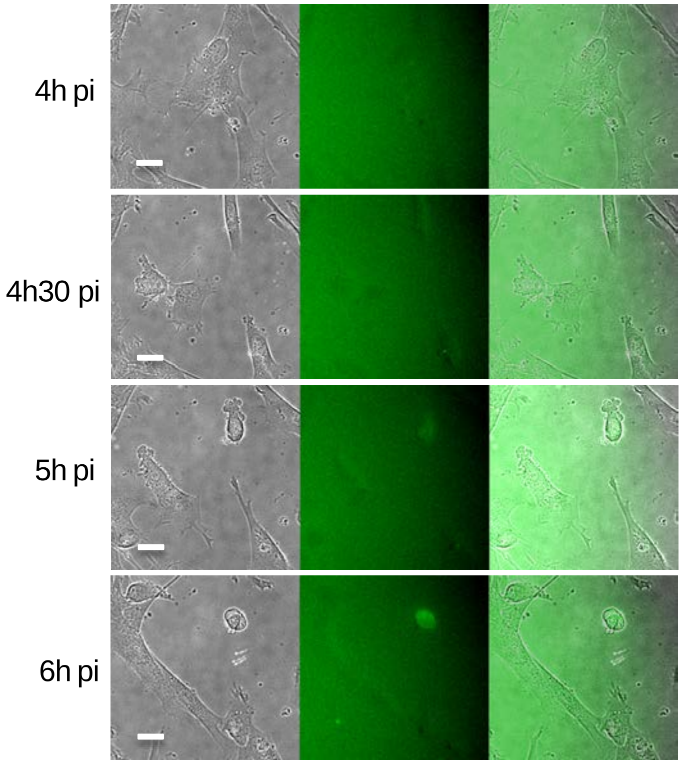

Figure S2. Initial demonstration of TB40-ANCHOR3 HCMV infection in MRC5 cells.
MRC5 cells were infected with TB40-ANCHOR3 HCMV viruses at an MOI of 0.5 and the first fluorescent cells could be detected between 4 and 5 hours pi. Infection induces rounding up of the cells which later turn back to their normal spindle shape (40X, scale bar 20µm);

Fig.S3

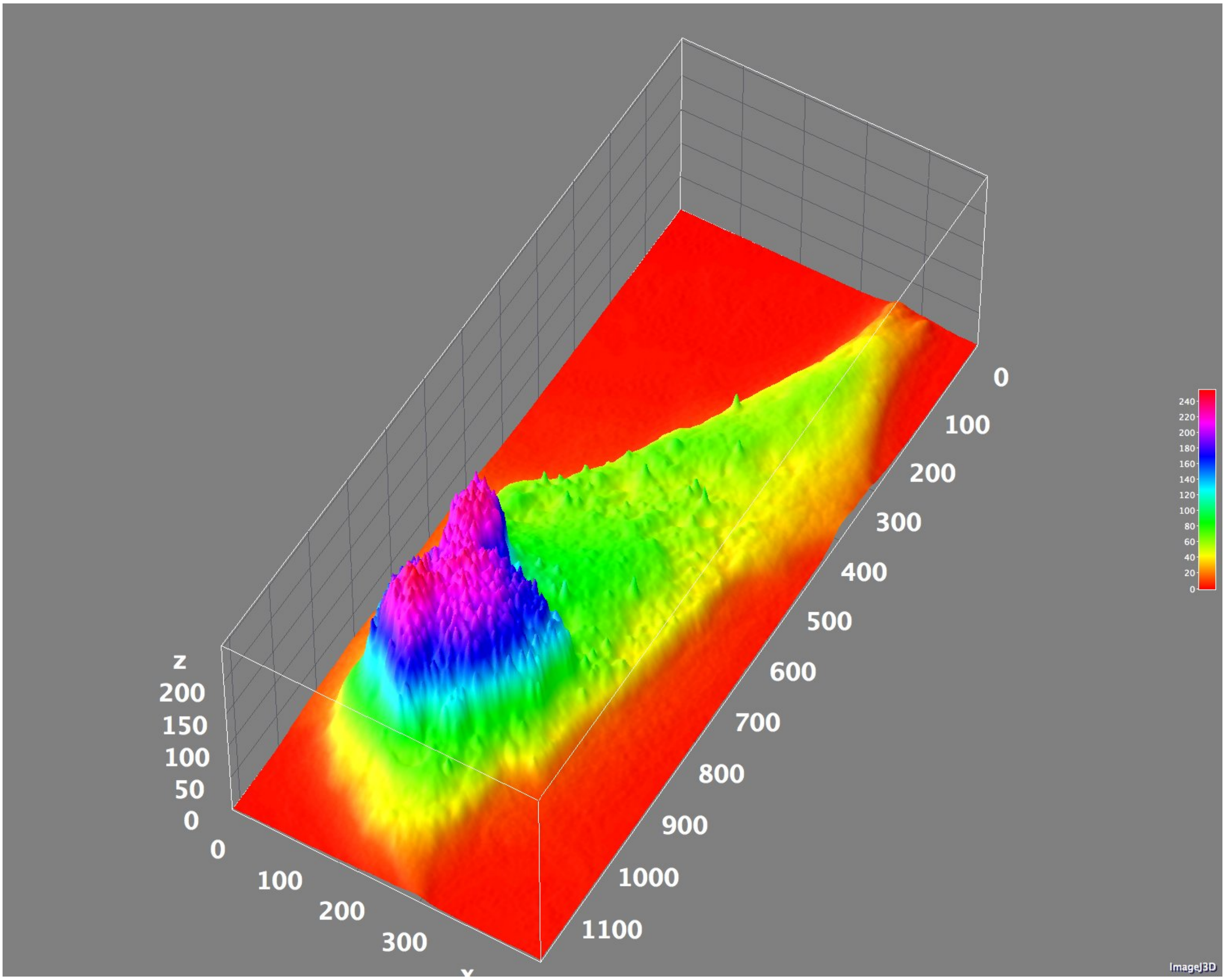

Figure S3. Visualization of viral replication in TB40-ANCHOR3 HCMV infected ARPE-19 cells. ARPE-19 cells were infected with TB40-ANCHOR3 HCMV at an MOI of 0.5 and analyzed 5 days pi. Quantification as in Fig.9c and d.

Fig. S4

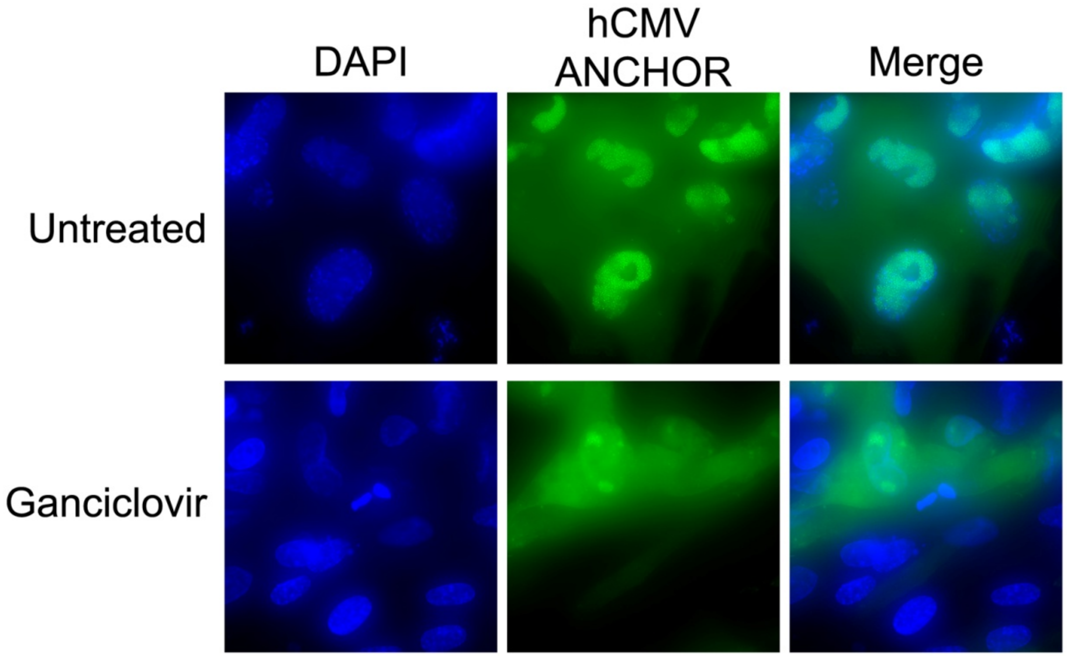

Figure S4. TB40-ANCHOR3 infected MRC5 cells, treated or not with 2.5µM Ganciclovir, were imaged 72h pi. In untreated cells, large RC are clearly visible at this stage of infection; on the contrary, only pre-replicative structures are observed at the same time in Ganciclovir treated cells.

Supplemental Material

Video 1: Time lapse imaging of MRC5 cells infected at a MOI of 1. Pictures were taken every 3h from 16h post infection to 97h pi on a wide-field Zeiss Axiovert, Observer Z1, 1.4NA objective, 40X.

Video 2: Time lapse imaging of MRC5 cells infected at a MOI of 0.5. Pictures were taken every 5 min from 63h pi to 66h pi on a wide-field Zeiss Axiovert, Observer Z1, 1.4NA objective, 63X. Apoptosis results in lysis which induces significant liberation of fluorescent material, possibly hCMV particles either alone or in vesicle, into the surrounding medium.

Video 3: Time lapse imaging of MRC5 cells infected at a MOI of 0.5. Pictures were taken every 5min from 48h pi to 67h pi on a wide-field Zeiss Axiovert, Observer Z1, 1.4NA objective, 63X. Example of lysis events by blebbing induced by hCMV ANCHOR.

Video 4: Time lapse imaging of ARPE-19 cells infected at a MOI of 1. Pictures were taken every 30min from 72h pi to 6 days pi on a wide-field Zeiss Axiovert, Observer Z1, 1.4NA objective, 63X. Example of infection to lysis event by blebbing induced by hCMV ANCHOR (upper cell left).
